# Phage display identifies Affimer proteins that direct calcium carbonate polymorph formation†

**DOI:** 10.1039/d4bm00165f

**Published:** 2024-07-24

**Authors:** Ilaria Sandei, Thembaninkosi Gaule, Matthew Batchelor, Emanuele Paci, Yi-Yeoun Kim, Alexander N. Kulak, Darren C. Tomlinson, Fiona C. Meldrum

**Affiliations:** a School of Chemistry, University of Leeds Leeds LS2 9JT UK F.Meldrum@leeds.ac.uk; b School of Molecular and Cellular Biology and Astbury Centre for Structural Molecular Biology, University of Leeds Leeds LS2 9JT UK

## Abstract

A key factor in biomineralization is the use of organic molecules to direct the formation of inorganic materials. However, identification of molecules that can selectively produce the calcium carbonate polymorphs calcite or aragonite has proven extremely challenging. Here, we use a phage display approach to identify proteins – rather than the short peptides typically identified using this method – that can direct calcium carbonate formation. A 1.3 × 10^10^ library of Affimer proteins was displayed on modified M13 phage, where an Affimer is a ≈13 kDa protein scaffold that displays two variable regions of 9–13 residues. The phage displaying the Affimer library were then screened in binding assays against calcite and aragonite at pH 7.4, and four different strongly-binding proteins were identified. The two aragonite-binding proteins generated aragonite when calcium and magnesium ions were present at a 1 : 1 ratio, while the calcite-binding proteins produce magnesium-calcite under the same conditions. Calcite alone formed in the presence of all four proteins in the absence of magnesium ions. In combination with molecular dynamics simulations to evaluate the conformations of the proteins in solution, this work demonstrates the importance of conformation in polymorph control, and highlights the importance of magnesium ions, which are abundant in seawater, to reduce the energetic barriers associated with aragonite formation.

## Abstract 2

A phage display approach is used to identify proteins – rather than the short peptides typically identified using this method – that can direct calcium carbonate formation, and demonstrates the importance of conformation in polymorph control.

## Graphical abstract

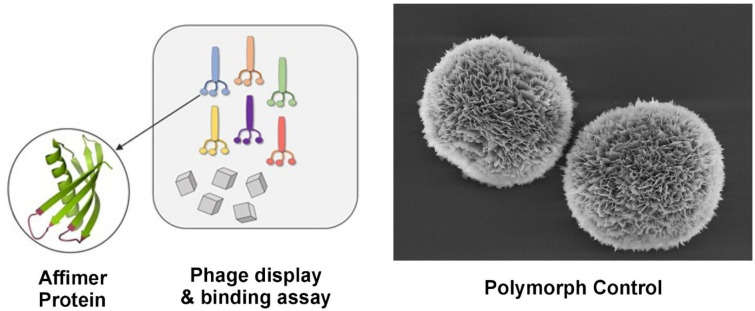

## Introduction

Evolution has enabled organisms to achieve astounding control over mineralization processes. This is seen in the formation of biominerals such as bones, seashells and fish-scales that are optimized for a wide range of functions including mechanical support, protection and even optical properties.^2^ In forming these structures, nature achieves a degree of control over crystallization that surpasses that achieved in synthetic systems, such as the generation of crystals with complex morphologies or specific polymorphs.^3^ This is principally achieved using organic molecules that can adopt roles ranging from soluble organic matrices to soluble additives. There has therefore been significant interest in translating these strategies to synthetic systems.

The calcium carbonate system, which precipitates as three anhydrous polymorphs under ambient conditions, provides one of the best examples of control over crystal polymorph in nature. Of these phases, calcite is the thermodynamically stable form at room temperature, aragonite is only slightly less stable, and vaterite is a kinetic polymorph.^4,5^ However, while both calcite and vaterite readily precipitate under ambient conditions, few synthetic organic additives have been identified that promote aragonite, and it is notable that all contain basic moieties.^6–8^ Yet, aragonite is a common biomineral, and some organisms such as mollusks can even switch between calcite and aragonite with perfect fidelity.^9^

Significant efforts have therefore been made to identify biomolecules that can select for calcite or aragonite, where biomolecules are extracted from aragonite and calcite biominerals and then employed as crystallization additives.^9–12^ This strategy has provided significant insight into the mechanisms by which organisms control calcium carbonate biomineralization, where the macromolecules are often highly acidic. However, there are few reports of the production of aragonite using proteins or protein fragments extracted from aragonite biominerals^13^ where most only generate aragonite when combined with more complex environments such as chitin substrates,^14,15^ an insoluble β-chitin/silk-fibroin matrix,^9^ or magnesium ions.^16^ Many questions therefore remain about the mechanisms by which organisms employ organic molecules to control polymorph formation.

One of the most effective approaches for the discovery of organic molecules that can direct the formation of inorganic materials is screening libraries of biomolecules. In most cases, this is conducted by expressing libraries of short peptides on phage or cells, and screening for their binding affinity to the target material. Application of this strategy to calcium carbonate has identified peptides that bind strongly to calcite, aragonite and vaterite, but which have only generated calcite or vaterite when they were employed in crystallization assays.^17–21^ Due to their small size, peptides lack the conformational rigidity of proteins that can lead to enhanced specificity in many scenarios.

**Fig. 1:**
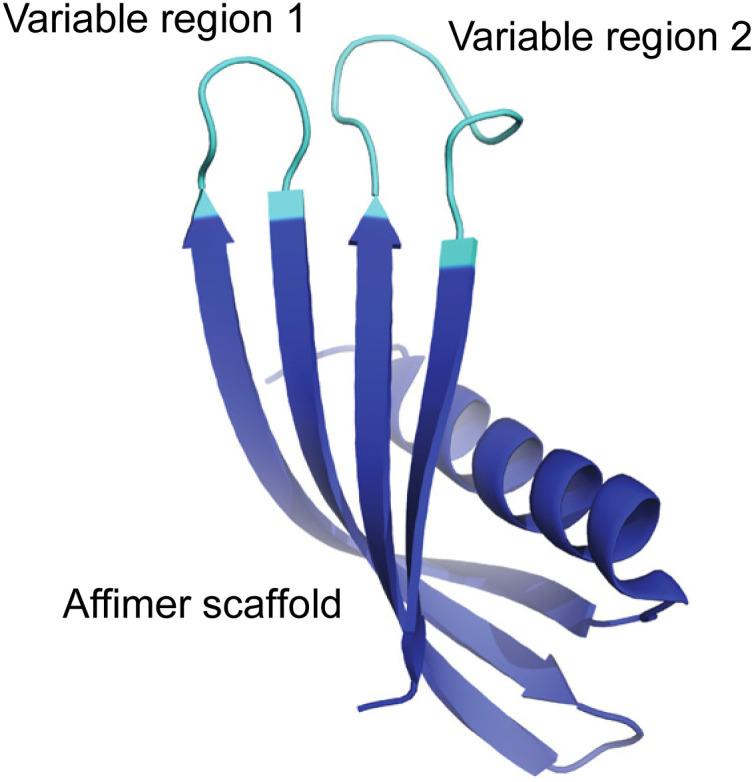
General representation of an Affimer protein. The two variable regions (or loops) are highlighted in cyan while the Affimer scaffold is highlighted in blue.

Here, we have used a phage display approach to screen libraries of proteins for their ability to direct the formation of calcite or aragonite, and thus explore the role of composition and conformational freedom on their activities. Affimer proteins were selected for this study, where these are small, stable recombinant proteins that possess two variable loops that can incorporate between 12 and 36 amino acids (Fig. 1). The protein scaffold constrains the possible conformations that the peptides in the loops can adopt, thereby distinguishing our approach from screens of phage-display libraries of short peptides. Screening of phage-display libraries of Affimer proteins has demonstrated strong binding to multiple targets.^22–24^

This was achieved by displaying Affimer proteins on the pIII minor coat protein of M13 phage, and screening for their ability to bind to calcite or aragonite. The most strongly-binding proteins are then expressed and employed in crystallization assays to determine their influence on calcium carbonate polymorph. Our experiments show that while polymorph selectivity is not achieved with the Affimer proteins alone, co-precipitation with low concentrations of magnesium ions enables the aragonite- and calcite-binding Affimer proteins to generate aragonite and calcite respectively. Magnesium ions are abundant in seawater^25^ and are expected to act in combination with biomolecules to select polymorphs in calcium carbonate biomineralization. Molecular dynamics (MD) simulations were used to evaluate the stability of the Affimer scaffold and the ensemble of conformations populated by the likely flexible variable loops. These data provide important insights into the role of the conformations of organic additives in achieving control over calcite/aragonite polymorphism.

## Results

### Design of experiments

Affimers are 12–15 kDa non-antibody synthetically engineered proteins that are based on the consensus sequence of cystatins (cysteine protease inhibitors). They exhibit monomeric structures and stability over a range of pH and temperature and do not possess glycosylation sites or disulfide bonds.^24,26^ Affimer proteins (originally called ‘Adhirons’) consist of two main regions: a scaffold comprising a 4-stranded antiparallel β-sheet and one α-helix, and two randomized peptide regions (often called loops or variable regions 1 and 2) of variable amino acid composition.^23,24,26^ (Fig. 1). The two variable regions are 9–13 amino acids in length and can bind to a target of interest, where their conformations are restricted by the protein scaffold. Randomization of the loop regions enables the construction of a large library (10^10^) of Affimer variants that can be employed in a phage display process to select high-affinity binder sequences towards small targets.^22–24^

**Fig. 2:**
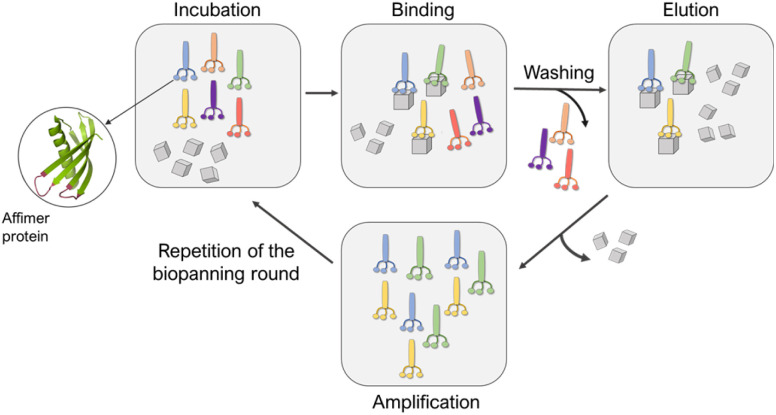
Schematic representation of the panning rounds performed against calcite and aragonite crystals.

A library of M13 phage encoded Affimer proteins can be generated by inserting the gene for the Affimer into the pIII gene of the minor coat protein of M13 phage. Subsequent transformation of the recombinant phagemid into *E. coli* cells followed by infection with a helper phage enables the production of phage particles displaying a specific Affimer variant on the pIII coat protein, with the two variable regions free to interact with small particles or other targets. The library used in our work was estimated to comprise 1.3 × 10^10^ individuals that vary in the amino acid composition of the variable loop regions, and are displayed on the pIII coat protein of M13 bacteriophage.^22,24,26^ Each phage therefore displays Affimer proteins that possess the same scaffold but differ in the compositions of the variable loops. The phagemid carries the genetic information to express that protein. Affimer proteins that bind strongly to calcite or aragonite are identified using a bio-panning process in which the Affimer phage library is incubated with calcite or aragonite crystals, respectively (Fig. 2). These “winning” proteins are sequenced to determine the composition of the variable regions. They are then expressed and used in crystallization assays to determine their influence on the polymorph and morphology of calcium carbonate.

### Selection of Affimer proteins

Affimer proteins that bind strongly to calcite or aragonite were identified by incubating the Affimer phage library with 70–160 nm calcite crystals synthesized using a carbonation method^27,28^ (Fig. S1†) or 50–100 μm long aragonite crystals synthesized at elevated temperatures^29^ (Fig. S2†). Briefly, calcite or aragonite crystals were (i) incubated with the Affimer phage library in a buffer solution at pH 7.4 at room temperature for 1 hour, then (ii) washed to remove unbound proteins, and finally (iii) dissolved to release the strongly-bound proteins. A total of four bio-panning rounds were performed, in each case starting with the individuals eluted from the previous round. The proteins isolated from the fourth round were then amplified and analyzed. As the phage DNA encodes for the specific Affimer protein displayed on the virion surface, the Affimer proteins can be identified by sequencing the corresponding phagemid. The sequencing was achieved by Genewiz® through the Sanger method.

### Sequences of Affimer proteins that bind to calcium carbonate

A total of 20 clones were selected after the fourth bio-panning rounds against both calcite and aragonite. The four most abundant clones were then selected for further investigation, where they were recombinantly expressed and tested in crystallization assays. DNA sequencing revealed that out of the 20 clones, two unique clones were selected that bound strongly to calcite (CBA-1 and CBA-2) and two that bound strongly to aragonite (ABA-1 and ABA-2). Although the Affimer phage library was designed to have two variable regions that are 9 amino acids long, CBA-2 has a 12 amino acid long variable region.

The distribution of amino acids across the variable regions of CBA-1 and CBA-2 were similar. Around 30% of the variable regions 1 and 2 comprise positively charged residues (R and K) which results in overall isoelectric points (pI) of 9.44 and 9.63 (Table 1) and an overall positive charge at pH 7.4. Whilst polar residues and hydrophobic residues make up 20% and 50% of the variable regions respectively, the Affimer proteins are expected to have high hydrophilicity (Table 1). Interestingly no negatively charged residues (D and E) were observed.

**Table 1:** Table summarizing the characteristics of the selected calcite-binding Affimer proteins (CBA-1 and CBA-2) and aragonite-binding Affimer proteins (ABA-1 and ABA-2)

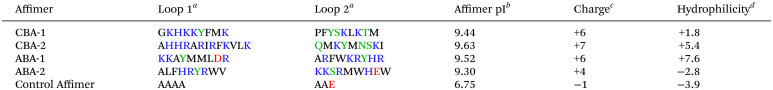

a Amino acids are color coded on the basis of charge at pH 7.4. Positively charged residues shown in blue; negatively charged ones in red; hydrophobic residues in black and polar residues shown in green.^54^

b Theoretical pI of Affimer protein obtained using the ExPASy ProtParam tool.

c Charge was calculated by subtracting the number of basic residues from the number of acidic residues, taking into account protonation at pH 7.4.

d Hydrophilicity was calculated using the Hopp and Woods index,^30^ where a positive value corresponds to hydrophilic and a negative value to hydrophobic.

The variable regions of the aragonite-binding Affimer proteins showed similar amino acid distributions to the calcium-binding Affimer proteins. However, the variable regions of both ABA-1 and ABA-2 do contain an acidic amino acid, namely D or E (Table 1). Although both Affimer proteins have a 50% distribution of hydrophobic residues within the variable regions and have an overall positive charge, ABA-2 is the most hydrophobic (Table 1) as it has more tryptophan residues.

### Probing the interactions of the selected Affimer proteins with calcite and aragonite

The specificity of the identified calcite- and aragonite-binding Affimer proteins for these crystal polymorphs was then investigated using a protein ELISA. A control Affimer protein was also generated to determine whether the scaffold itself binds to the calcium carbonate crystals. This has very short variable regions comprising four alanines in the first loop and two alanine and one glutamic acid in the second loop (Table 1). This test was conducted by exposing the proteins to equal amounts of the individual polymorphs for 1 hour in casein blocking buffer, and then washing the crystals to remove unbound proteins. After the addition of Anti-His antibodies that bind to the His-tag on the Affimer proteins, and subsequent addition of TMB (3,3′,5,5′-tetramethylbenzidine), the color of the solution changes to light blue if Affimer proteins are bound to the crystals. This was quantified by measuring the absorbance of the solution at 620 nm. No color change was observed after incubating calcite or aragonite with the Affimer control proteins, which demonstrates that binding of the proteins to the crystals is mediated through the variable loop regions.

**Fig. 3:**
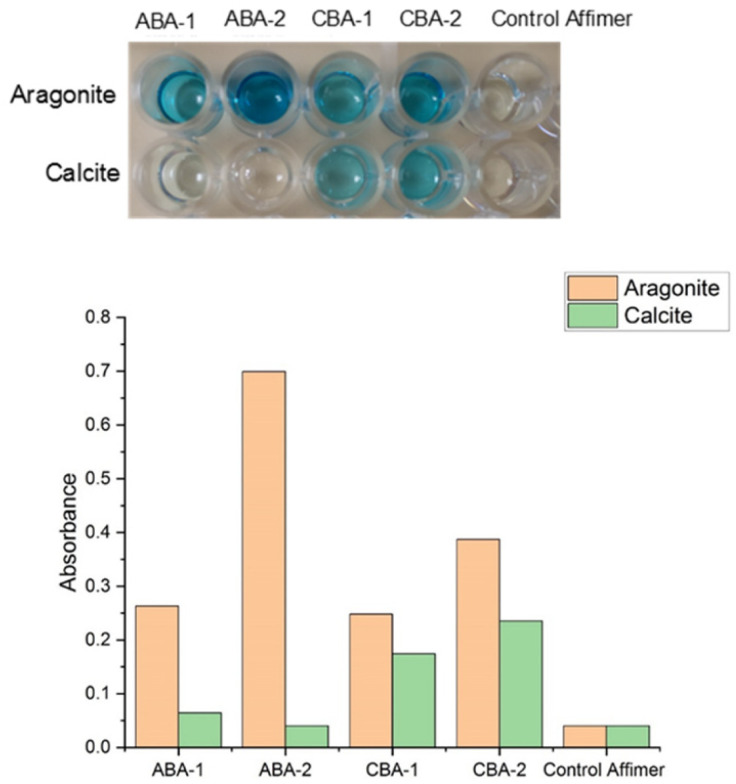
ELISA of the aragonite- and calcite-binding Affimer proteins and the control Affimer protein against calcite and aragonite, and absorption measurements taken at 620 nm. The blue color is indicative of binding. The ABA-1 and ABA-2 proteins only bind to aragonite, while the CBA-1 and CBA-2 bind to both aragonite and calcite, with a slightly higher affinity for aragonite. The scaffold, as represented by the control Affimer protein, binds to neither polymorph, confirming that the binding of the proteins to the crystals is mediated through the loop regions.

The ELISAs of the ABA and CBA proteins confirmed that all of these proteins bind to the polymorphs used in the bio-panning rounds. However, while ABA-1 and ABA-2 only bind to aragonite and have no affinity for calcite, CBA-1 and CBA-2 bind to both polymorphs, albeit with a slightly higher affinity for aragonite. This was indicated by the lack of color in the solution of ABA-1 and ABA-2 incubated with calcite, and an intense blue color when ABA-1, ABA-2, CBA-1 or CBA-2 were incubated with calcite or aragonite targets (Fig. 3).

### Calcium carbonate precipitation in the presence of the selected Affimer proteins

Crystallization experiments were then conducted to investigate the influence of the identified CBA and ABA proteins on calcium carbonate crystallization. The Affimer proteins were expressed as described previously.^24^ They were then purified using Ni-NTA resin chromatography and buffer exchanged into H_2_O to remove phosphate ions or buffers that could affect the crystallization. SDS-PAGE analyses demonstrated that the selected Affimer proteins were expressed with good yields and purities (Fig. S3†).

**Fig. 4:**
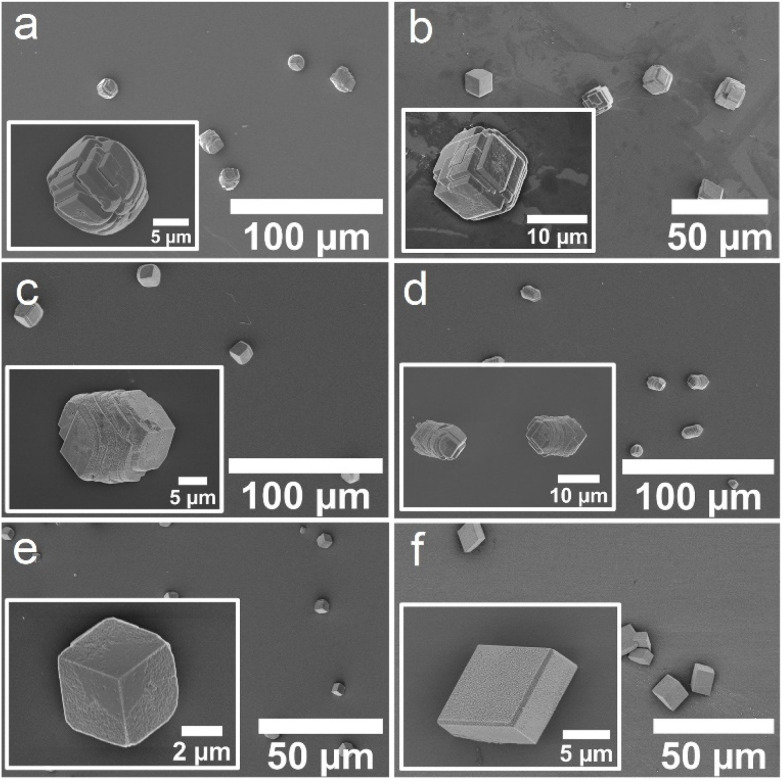
SEM micrographs of crystals obtained using the ADM method in the presence of (a) ABA-1, (b) ABA-2, (c) CBA-1, (d) CBA-2, (e) the control Affimer protein, and (f) in the absence of any protein.

Calcium carbonate was precipitated in the presence of these Affimer proteins using the ammonia diffusion method (ADM),^31^ in which solutions comprising [CaCl_2_] = 2 mM and [Affimer] = 10 μM were exposed to ammonium carbonate vapor in a sealed chamber. Crystals formed on glass slides placed at the base of the reaction solution and were subsequently removed for characterization. Calcite formed in the presence of all four ABA and CBA proteins and the control Affimer protein (Fig. 4a–f).

Characterization of the crystals using scanning electron microscopy (SEM) showed some morphological differences; crystals formed with ABA-1 and ABA-2 were rhombohedral in form with blocky steps on the {104} faces (Fig. 4a and b), while those grown in the presence of CBA-1 and CBA-2 were elongated along the *c*-axis (Fig. 4c and d), which is indicative of preferential binding of the proteins to the acute over the obtuse steps on the {104} faces.^27,32^ CBA-2 induced slightly greater elongation, and in both cases the equatorial zone featured macroscopic, blocky steps that derive from step bunching. Control experiments performed with the control Affimer protein yielded rhombohedral calcite crystals with slightly roughened surfaces (Fig. 4e), while those conducted in the absence of protein yielded 10–20 μm rhombohedral calcite crystals with smooth faces (Fig. 4f).

**Fig. 5:**
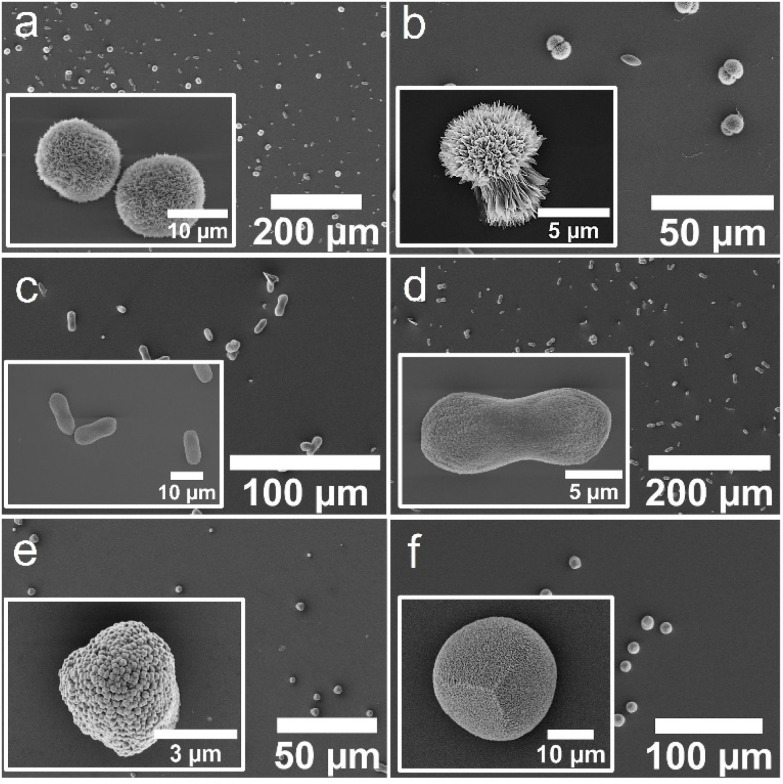
SEM micrographs of crystals obtained using the ADM method in the presence of Mg^2+^ ions at a Ca : Mg ratio 1 : 1 and (a) ABA-1, (b) ABA-2, (c) CBA-1, (d) CBA-2, (e) the control Affimer protein, and (f) in the absence of any protein. The images shown in the insets are (a and b) aragonite crystals and (c–f) calcite crystals.

The influence of the Affimer proteins on calcium carbonate precipitation was also explored in the presence of magnesium ions, since magnesium ions are abundant in seawater^25^ and are expected to play an important role in calcium carbonate biomineralization. Notably, magnesium ions are recognised to promote the formation of aragonite at room temperature in certain concentration regimes.^33–35^ Experiments were conducted at [CaCl_2_] = [MgCl_2_] = 2 mM, where calcite forms in the absence of Affimer protein (Fig. 5f).

**Fig. 6:**
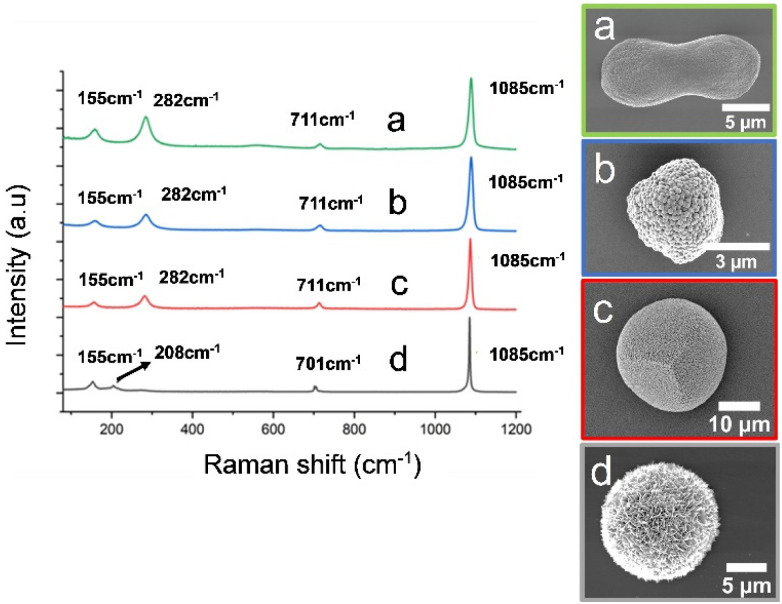
Raman spectra of crystals obtained from the different experiments conducted in the presence and absence of proteins: (a) calcite crystals obtained using ADM in the presence of Mg^2+^ ions at a Ca : Mg ratio 1 : 1 and CBA proteins, (b) calcite crystals obtained using ADM in the presence of Mg^2+^ ions at a Ca : Mg ratio 1 : 1 and the control Affimer protein, (c) calcite crystals obtained using ADM in the presence of Mg^2+^ ions at a Ca : Mg ratio 1 : 1 and in the absence of any protein, (d) aragonite crystals obtained using ADM in the presence of Mg^2+^ ions at a Ca : Mg ratio 1 : 1 and ABA proteins. All of them show the characteristic peak at 1085 cm^−1^ related to the symmetric stretching vibration of the carbonate group while the peak of the in-plane bending of the CO_3_^2−^ group shifts from (a–c) 711 cm^−1^, to (d) 701 cm^−1^. For (d) the characteristic peak of calcite at 282 cm^−1^ is absent while we see the peaks at 155 cm^−1^ and 208 cm^−1^ related to the translation and libration modes of the carbonate ions in aragonite.

Near identical magnesium-calcite crystals with peanut-like morphologies formed in the presence of both CBA proteins (Fig. 5c and d), and the polymorph was confirmed by Raman spectroscopy (Fig. 6). In contrast, the ABA proteins both yielded aragonite as the principal phase (Fig. 5a and b), where the crystals were spherical (ABA-1) or resembled overgrown dumbbells and comprised bundles of needles (ABA-2). 60% aragonite and 40% Mg-calcite formed in the presence of ABA-1, while ABA-2 generated 90% aragonite and 10% Mg-calcite crystals. The Mg-calcite crystals formed in these experiments were lozenge-shaped.

Control experiments performed with the control Affimer protein yielded calcite crystals with blocky steps (Fig. 5e), while those conducted in the absence of protein yielded near-spherical calcite crystals with a roughened surface (Fig. 5f). Additional transmission electron microscopy (TEM) analyses were performed on the Mg-calcite and aragonite crystals after preparing thin sections using focused ion beam (FIB) milling. This demonstrated that the Mg-calcite were single crystals, and the aragonite was polycrystalline; polymorphs were confirmed using selected area electron diffraction (SAED) (Fig. S4 and S5†).

### Calcium carbonate precipitation in the presence of peptides

The Affimer proteins provide a scaffold that limits the conformational freedom of the variable loops. The role of the conformation of the loops in their ability to interact with calcium carbonate surfaces was explored by precipitating calcium carbonate in the presence of 10 μM peptides with the same compositions as the variable loops. The peptides investigated are summarized in Table S1.† Calcite alone was obtained in the presence of all of the peptides tested, both in the presence and absence of Mg^2+^ ions. Representative data are given in Fig. S6–S9† for peptides with the same sequences as loops 1 and 2 from CBA-1. Addition of Mg^2+^ ions resulted in somewhat less regular morphologies, and increased surface roughness (Fig. S7 and S8†). In the absence of Mg^2+^ ions, perfect calcite rhombohedra formed in the presence of loop 1 peptides, loop 2 peptides or both loop 1 and 2 peptides (Fig. S8†). These results suggest that the constrained conformation adopted by the peptides within the Affimer scaffold is crucial to their interaction with the crystals.

### Molecular dynamics (MD) simulations

**Fig. 7:**
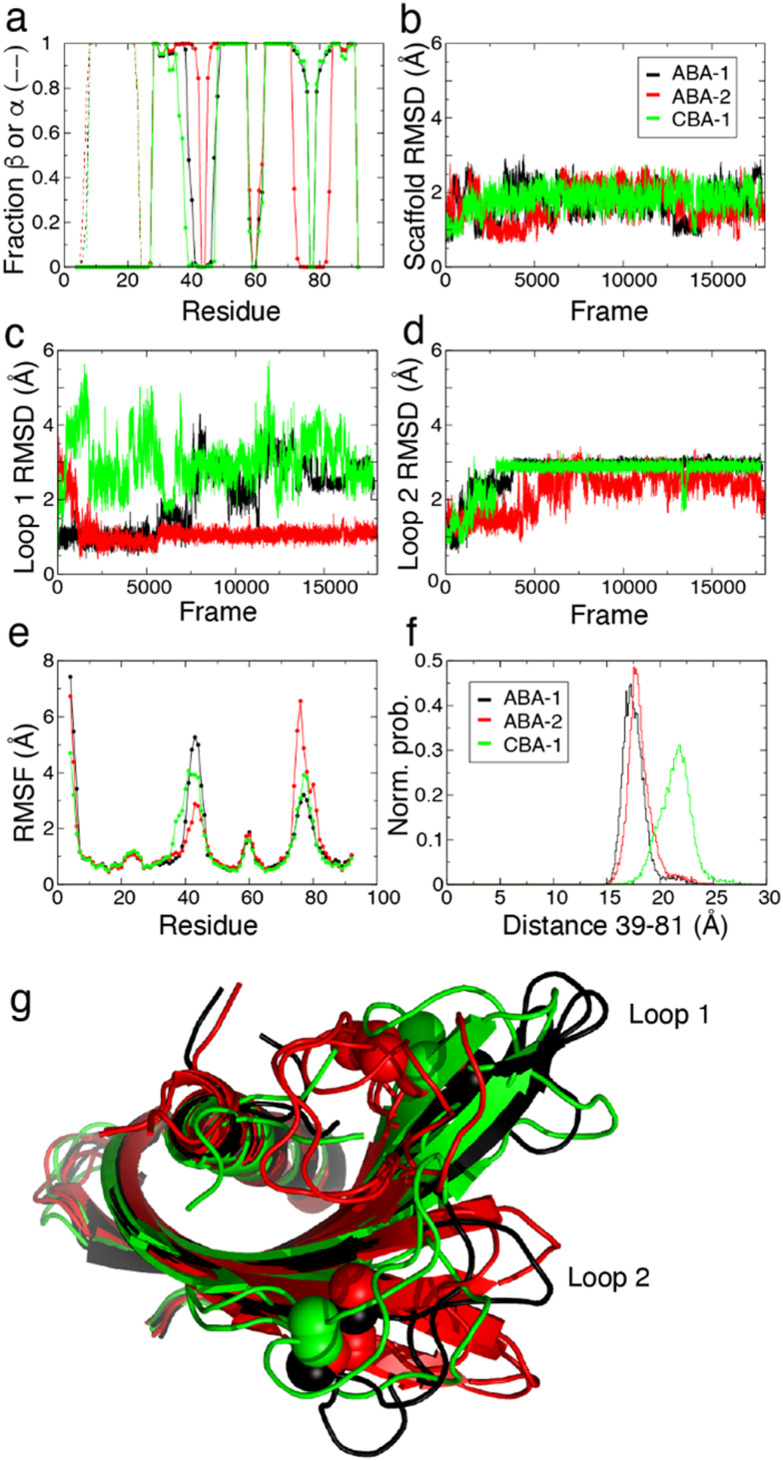
Molecular dynamics simulations of Affimers: ABA-1 (black), ABA-2 (red) and CBA-1 (green). (a) Secondary structure analysis on a per residue basis averaged over the simulation; dotted lines show the position of the α helix, solid lines and circles show the positions of the β strands. (b) RMSD of Cα atoms with respect to the initial structure of Affimer scaffolds (residues 4–38, 48–72, 82–92) over the course of the simulation. (c) Cα RMSD of loop 1 (residues 39–47). (d) Cα RMSD of loop 2 (residues 73–81). (e) RMSF values for the three simulations. (f) The distance between the outer edges of the two loops as gauged by the Cα to Cα distance for residues 39 and 81. (g) Representative structures from major clusters (>10% occupancy, accounting for >70% of the simulation in total) observed in the three simulations. Three major clusters were observed for ABA-1 (28, 26, 20%) and CBA-1 (49, 12, 11%), four major clusters were observed for ABA-2 (31, 26, 20, 14%).

MD simulations were used to model the conformations adopted by the Affimer scaffold and the variable loops of CBA-1, ABA-1 and ABA-2 in solution environments. These three Affimer proteins have the same scaffold sequence and loops of the same length as an Affimer for which there is a published X-ray crystal structure (PDB code: 6YXW).^36^ CBA-2 has longer loops and so was not modelled. Initial structures for simulation of CBA-1, ABA-1 and ABA-2 were built by mutating the loop residues of the 6YXW model to match the required sequence (Table 1). The simulations show that the scaffold is structurally stable in all cases. The secondary structure motifs are an α-helix (residues ≈8–22) and four β-strands (residues β1 ≈ 28–35, β2 ≈ 49–57, β3 ≈ 63–71 and β4 ≈ 84–91) and all were highly conserved in the simulations (Fig. 7a). There were some differences in the observed length of the β strands, however, where loop 1 contributed to a longer pair of strands (β1 and β2) in ABA-2, while loop 2 contributed to a longer pair (β3 and β4) in ABA-1 and CBA-1.

The root mean square deviation (RMSD) describes the variation in the structure of the protein over time.^37^ For the scaffold—*i.e.* all residues except the two variable loops (residues 39–47 and 73–81)—the RMSD values are low at 1–2 Å. This shows that the Affimer scaffolds have similar structures in solution to the 6YXW crystal structure (Fig. 7b). As expected, the RMSD values for the variable loop regions (Fig. 7c and d) are generally higher than for the scaffold, demonstrating some deviation from the loop structures of 6YXW (which has different loop sequences). RMSD values are particularly high for loop 1 in the CBA-1 Affimer. This reflects a change in structure as compared with the initial structure, and could result from the adoption of an alternative static structure and/or an increase in the dynamics or flexibility of the protein or protein region.

Root mean square fluctuation (RMSF) values describe, for each residue in the sequence, the average deviation of that residue away from its simulation average position. The RMSF therefore presents a simple metric for comparing more stable regions of a protein with areas that are more flexible or dynamic. As can be seen from the plots of RMSF for the three Affimer proteins (Fig. 7e), the protein regions with secondary structure have low flexibility whereas the N-terminus and loops—and particularly the two variable loops—show higher variability. Interestingly, ABA-1 and ABA-2 both have one very flexible loop and one more constrained loop, while CBA-1 has two intermediately flexible loops. The flexibility of loop 1 for CBA-1 also extends further towards the N-terminus than the aragonite-binding Affimers, reducing the effective length of the first β strand (see Fig. 7a). As a result, the gap between the outer edges of the four strands making up the β sheet (or the edges or ‘reach’ of the two loops, as described by the distance between residues 39 and 81) is larger in the case of CBA-1 compared to the two aragonite-binding Affimers (Fig. 7f).

Further insight can be gained by identifying similar conformations seen in the simulations (based on relative RMSD values) and comparing representative structures for each of these clusters for each of the different Affimer proteins. Equivalent cluster analyses gave a total of nine clusters for ABA-1 and ten clusters for both ABA-2 and CBA-1, and reversible interconversion between conformations belonging to different clusters was observed (Fig. S10†). Representative structures for the most highly populated clusters (observed for >10% of the simulation) are shown with the scaffolds overlaid in Fig. 7g. The structures overlay very well away from the loop regions, although two different positions are observed for the β3/β4 hairpin for all Affimer proteins. This is seen most clearly for ABA-2 where the hairpin is longest and loop 2 shortest (Fig. 7g). The representative cluster structures clearly show a range of preferred loop conformations, where loop 1 conformations are folded closer to the helix in ABA-2, and CBA-1 has loop 2 conformations closer to the helix. The simulations therefore reveal a range of conformational preferences around the variable loop regions for the three different Affimers, which although difficult to deconvolute, could go some way to explain preferred binding to the different calcium carbonate polymorphs.

## Discussion

Phage display techniques have been widely used to identify peptides that bind strongly to biological and inorganic substrates.^18,21,22,38–43^ At the heart of this strategy is the ability to display, and then subsequently screen vast combinatorial libraries of peptides against the chosen target. Multiple screening rounds identify the peptide sequences that form the strongest interactions with the target, and the inherent link with the genotype provided by the phage enables the individual “winning” peptides to be sequenced and then expressed. The effect of the specific sequences on mineral formation can then be studied in scaled-up experiments.

A number of previous studies have used phage display to identify peptides that bind strongly to the individual calcium carbonate polymorphs.^17,18,20,21,44^ The protein sequences identified have many similarities to those found in the current study, in which basic and hydrophobic amino acids were prevalent. Schüler *et al.* identified two heptamers following three bio-panning rounds against vaterite crystals that only contained hydroxylic and hydrophobic residues, of which proline was the most abundant.^20^ Vaterite formed in crystallization assays using one of these heptamers, while calcite formed when glycine had been substituted for proline in the same peptide. Gaskin *et al.* identified 10-mer peptides that bound strongly to calcite or aragonite and again showed that these were not rich in acidic amino acids, where they contained just one Glu or Asp each.^18^ Li *et al.* screened a 15-mer peptide library against calcite crystals, and identified nine strongly-binding peptides that were typically rich in hydroxylated (Ser and Thr) and hydrophobic residues.^17^ Gebauer *et al.* studied the influence of 12-mer peptides – identified as strong binders to geological aragonite – on calcium carbonate crystallization.^44^ The three peptides studied were basic, and all inhibited calcite formation but did not generate aragonite. Finally, Völkle *et al.* compared the effects of mixtures of proteins extracted from sea urchin skeletal plates with 12-mer peptides identified in binding assays of phage libraries to calcite and aragonite at pH 7.5.^21^ Both sets of peptides exhibited a broad range of isoelectric points and were depleted in negatively-charged residues, while the aragonite-binders were enriched in lysine and histidine. However, calcite was the only polymorph produced in all crystallization experiments.

It is valuable to compare the sequences of these peptides with those of proteins found associated with calcite and aragonite biominerals. Proteins associated with the aragonite nacreous layer of mollusks can be acidic or basic. The acidic matrix protein Pif has been widely investigated,^10,14,15,45^ and the C-terminal (Pif80) and N-terminal regions (Pif97) appear to have different roles in aragonite formation.^10,14,45^ Both regions are highly acidic with pI values of 4.99 and 4.65 respectively, but Pif80 has a higher percentage of charged residues and exhibits a highly repetitive four-residue motif (Asp–Asp–Arg(Lys)–Lys(Arg)) and a cluster of acidic amino acids (Asp–Glu–Asp).^14,15^ It has been suggested that Pif97 may bind to the chitin layer *in vivo*, while Pif80 may be involved in controlling the nucleation and orientation of aragonite crystals.^45^ Proteins that are rich in basic residues are also believed to contribute to aragonite formation *in vivo*: KRMP-3,^46^ N40,^47^ PfN23,^48^ Prisilkin-39^49^ and PNU9.^50^ Proteins of the KRMP family include a lysine-rich basic region and a Gly/Tyr-rich region,^46^ which is comparable to the Affimer proteins selected here for binding to aragonite.

Proteins found in the prismatic layer of mollusk shells are often rich in glycine and aspartic acid.^46,51,52^ MSP-1,^52^ aspein^51^ and asprich^53^ are examples of proteins with high aspartic acid contents, while MSI31,^54^ MSP-1^52^ and prismalin-14^55^ are glycine-rich, featuring GGY domains. MSI31 has 10 poly(glycine) repeats of 3–5 residues and six acidic motifs principally comprising Glu residues.^56^ The glycine-rich region may participate in the formation of β-sheet structures, while the acidic domain would enable binding of Ca^2+^ ions.^56^ MSP-1, in contrast, is an acidic glycoprotein with -SG-, D and K rich domains.^52^ Calprismin and Caspartin, two proteins extracted from the prismatic layer of *Pinna nobilis*, are mainly acidic, where Ala, Asx, Thr, and Pro dominate the amino acid composition of calprismin, and Asp is the main residue in Caspartin.^57^ In contrast, the shell matrix proteins (shematrins) identified in the pearl oyster *Pinctada fucata* and associated with the formation of the calcitic prismatic layer possess repetitive basic RKKKY, RRKKY or RRRKY domains at their C-termini.^58^

These data highlight the challenge and complexity of exploring the link between protein/peptide composition and calcium carbonate polymorph, where comparison of all of these studies suggests that there is no stand-out distinction between calcite- and aragonite-binding molecules. In an attempt to identify patterns, a recent study^59^ used machine-learning to analyse the compositions of calcite and aragonite-binding 12-mers identified in the study by Völkle *et al.*^21^ This revealed some differences for residue location within the peptides, and suggested that most difference occurred for ‘tiny’, aliphatic, aromatic, acidic and basic residue descriptors.

This body of work, together with our observations that the compositions of the Affimer proteins selected in the calcite and aragonite binding assays are similar (where all principally comprise basic and hydrophobic residues) strongly suggests that the amino acid compositions of the peptides and proteins are not the sole determinant in biological control over calcium carbonate polymorph. The main differences are the presence of a single acidic residue in the ABAs, and the principal amino acid in the ABAs is arginine rather than lysine in the CBAs. Modelling studies of the interactions between a calcite surface and the primary (–NH_2_) amine in the Lys side chain and the secondary amine of the Arg side chain have shown that they interact strongly with the carbonate anions at all adsorption sites.^6^ The amines are positively charged under the experimental conditions, and Lys binds more strongly than Arg. No comparable data exist for aragonite.

This indicates that the conformations and conformational freedom of the proteins are also crucial in determining their interactions with the crystal surface, and their ability to direct calcium carbonate crystallization. In this way, the hydrophobic residues, which do not bind directly to the crystal surface but do dictate the conformation of the proteins, play important roles in determining the protein behavior. Changes in the conformations of Affimers close to the variable loop regions were clearly shown here using MD simulations, and these differences will likely account for how the proteins interact with the crystal surface. This is also supported by the studies conducted with peptides of composition identical to those of the loop regions; calcite formed rather than aragonite in the presence of peptides with ABA protein loop sequences. The constraints imposed on the peptides by the Affimer scaffold is therefore important in defining the interactions between the loops and the crystal surface.

Finally, our study highlights the likelihood that magnesium ions may act in combination with biomolecules to direct calcium carbonate crystallization. Few proteins or protein fragments^13^ direct aragonite formation without combining them with more complex environments such as chitin substrates,^14,15^ an insoluble β-chitin/silk-fibroin matrix,^9^ or magnesium ions.^16^ The addition of magnesium ions is one of the most robust methods of generating aragonite under ambient conditions, where they inhibit the formation of calcite, allowing magnesium-calcite and ultimately aragonite to form as the Mg/Ca ratio in solution increases.^34,60,61^ Given the high concentration of magnesium ions in seawater, their combination with biomacromolecules could offer a straightforward means of tuning crystal polymorph *in vivo*.^6,62^

## Conclusions

In summary, this article demonstrates the use of phage display to screen a combinatorial library of Affimer proteins and identify individual proteins that bind strongly to calcite and aragonite. As compared to randomized peptides that have previously been selected as crystallization additives using phage display,^17,18,20^ proteins offer the possibility of constraining the conformations of the variable loops, and provide easier genetic manipulation of the phagemid for protein production.^22,24^ Affimer proteins are particularly well suited to this approach, since they are stable under a wide range of conditions.^23,24,26^ While polymorph selectivity was not achieved in the presence of the proteins alone, the aragonite-binding Affimer proteins generated aragonite when low concentrations of magnesium ions were present, and the calcite-binding proteins generate calcite. Notably, peptides with sequences corresponding to the variable loops of the aragonite-binding proteins only formed calcite. The conformations of Affimer proteins were shown to differ close to the variable loop regions in dynamic molecular models of the aragonite- and calcite-binding proteins. These results demonstrate that the conformation of the organic additives can play a significant role in determining their interaction with growing crystals, and also highlight the value of additives acting in combination with magnesium ions to direct calcium carbonate formation *in vivo*. Indeed, lower concentrations of magnesium ions are required to generate aragonite when these organic molecules are present, giving the advantage of faster crystallization rates than are achieved at high Mg/Ca ratios. Further work will use simulations to study the interactions of the Affimer proteins with calcite and aragonite surfaces.

## Author contributions

IS carried out the majority of the experimental work and characterisation. TG assisted and provided guidance with the molecular biology studies, while YYK provided guidance with the crystallisation work. AK assisted with SEM. MB and EP led the simulations studies, with assistance from IS. FCM and DT supervised the project. The manuscript was written through contributions of all authors and all authors have given approval to the final version of the manuscript.

## Data availability

The data that support the findings of this study are openly available in the Research Data Leeds Repository at https://doi.org/10.5518/1374.^1^

## Conflicts of interest

There are no conflicts to declare.

## Supplementary Material

BM-012-D4BM00165F-s001
