# Supplementary material for: Phage display identifies Affimer proteins that direct calcium carbonate polymorph formation
Source: Biomater Sci. 2024 Jul 24;12(20):5215–24. doi: 10.1039/d4bm00165f (PMC11358866; doi:10.1039/d4bm00165f)
Supplement: BM-012-D4BM00165F-s001 [file BM-012-D4BM00165F-s001.pdf]

Supplementary Information (SI) for Biomaterials Science.
This journal is © The Royal Society of Chemistry 2024

**SUPPLEMENTARY INFORMATION**

# Phage Display Identifies Affimer Proteins that Direct Calcium Carbonate Polymorph Formation

*Ilaria Sandei, Thembaninkosi Gaule, Matthew Batchelor, Emanuele Paci, Yi-Yeoun Kim, Alexander N. Kulak, Darren Tomlinson and Fiona C. Meldrum*

## 1. Materials

### 1.1 Stock Solutions

All the buffers were filtered with Durapore® Membrane Filters 0.22 µm prior to use

PBS (Phosphate buffered saline) (NaCl 137 mM, KCl 2.7 mM, $Na_2HPO_4$ 10 mM, $KH_2PO_4$ 2 mM, dd$H_2O$, pH 7.4)

PBST (PBS supplemented with 0.1% Tween-20)

Tris-HCl (Trizma® 1 M, HCl, pH 7 in dd$H_2O$)

Tris-HCl (Trizma® 1 M, HCl, pH 9 in dd$H_2O$)

Glycine 0.2 M (Glycine in dd$H_2O$)

Wash buffer ($NaH_2PO_4$ 50 mM, NaCl 500 mM, Imidazole 20 mM, pH 7.4)

Wash solution ($H_2O$, 20 mM Imidazole)

Elution solution ($H_2O$, 300 mM Imidazole)

LB broth (LB Broth base Invitrogen™ in dd$H_2O$ according to manufacturer's instructions)

IPTG (1M stock solution in dd$H_2O$)

Carbenicillin (500x stock: 50 mg/mL in dd$H_2O$)

Kanamycin (500x stock: 25 mg/mL in dd$H_2O$)

2TY medium (10 g yeast extract, 16 g tryptone, 5 g NaCl per 1 L of dd$H_2O$)

## 2. Methods

### 2.1 Synthesis of Calcite and Aragonite for Bio-panning

Nano-calcite was precipitated following the carbonation method.[1] The use of carbonation rather than the ammonia diffusion and Kitano methods was preferred due to the possibility of obtaining pure, nanosized calcite crystals (significant amounts of aragonite and vaterite were also observed with the other methods) with good yields. This was required to enable the biopanning rounds using a phage display library. For the process, 0.44 g of CaO in 60 mL of DI $H_2O$ (already degassed for 6 hours at 90 °C) were stirred at room temperature under a $N_2$ atmosphere for ~ 12 hours and then, a gas flow of $N_2$:$CO_2$ = 3:1 was provided until the achievement of a pH 7.22. The crystals obtained were washed with MilliQ water and ethanol three times and dried and characterised using SEM and Raman spectroscopy. The crystals were highly monodisperse with rhombohedral shape and sizes between 70 and 160 nm (Figure S1A). Characterization of the crystals using Raman spectroscopy demonstrated that they were calcite (Figure S1B).

Aragonite was synthesized using the method described by Wang et al.[2] Briefly, a solution of 0.25 M $CaCl_2{\cdot}2H_2O$ (Sigma-Aldrich) and 0.75 M urea (Sigma-Aldrich) was incubated at 75 °C for 16 hours. The crystals were washed with MilliQ water and ethanol three times and dried and characterised using SEM and Raman spectroscopy. The orthorhombic aragonite crystals obtained had sizes of between 100 and 200 μm. (Figure S2A). Characterization of the crystals using Raman spectroscopy demonstrated that they were aragonite (Figure S2B).

## 2.2 Phage Display Methodology

Affimer proteins that bind strongly to calcite or aragonite were identified using a phage display method, performing in total four bio-panning rounds of selection, as described by Tiede et al.[3] with some adjustments. Briefly, 5 μL of a phage library comprising $1.3x10^{10}$ clones was mixed with 245 μL of Blocking Buffer Solution (made by mixing 10 mL of Casein Blocking Buffer

10X and 40 mL of PBST). This solution was exposed to ≈ 1 mg of nanosized calcite crystals (~100 nm in size) for 1 hour at room temperature. Then unbound phage were removed from the crystals by addition of fresh PBST buffer (137 mM NaCl, 2.7 mM KCl, 10 mM $Na_2HPO_4$, 2mM $KH_2PO_4$, pH 7.4, 0.1% Tween 20), centrifugation (8000 rpm, 3 min, R.T), and removal of the supernatant solution with its replacement with fresh 500 µl of PBST. This extraction process was repeated 12 times.

The phage bound to the calcite crystals were then eluted by adding to the calcite crystals 100 µL of glycine–HCl (0.2 M, pH 2.2) and leaving the solution at R.T for 10 min, with partial dissolution of the calcite crystals. 15 µL of Tris–HCl (1 M, pH 9.1) were then added, and the supernatant solution was mixed added to 8-mL of ER2738 cells culture in 2TY medium ($OD_{600}$ = 0.2). The remaining phage were then eluted by adding to the calcite 100 µl of triethylamine and leaving the solution at R.T for 6 min. The solution was then neutralized with 50 µl of 1 M Tris-HCl (pH 7) and the entire supernatant was then added to the same ER2738 cells culture. The cells were incubated at 37 °C for 1 hour. 100 µL of the cell culture solution were then plated on LB carb plates (LB agar plates supplemented with 100 µg $mL^{-1}$ carbenicillin) and incubated overnight at 37 °C.

The cells were scraped from the plate using 8 mL of a solution of 2TY medium and 100 µg $mL^{-1}$ of carbenicillin and grown for 1 hour (37 °C, 230 rpm) to an $OD_{600}$ of 0.2. 0.32 µL of M13KO7 helper phage (titre ca. $10^{14}$ $mL^{-1}$) was added to the solution and incubated (37 °C, 90 rpm, 30 min). 16 µL of kanamycin (50 µg $mL^{-1}$) was added at room temperature and the solution was incubated overnight (170 rpm, 25 °C). The solution containing the phage-infected cultures was then centrifuged (3500*g*, 10 min) and 125 µL of the supernatant (which contains phage) was used to perform another panning round. The entire procedure just described was

performed a total of 4 times in order to achieve a greater selectivity in isolating the calcite-binding Affimer proteins.

The same method was repeated using ≈ 1 mg of aragonite in place of the calcite in order to isolate Affimer proteins that bind strongly to aragonite.

## 2.3 DNA Purification and Sequencing

The DNA coding for the strongly-binding Affimer proteins was then purified and sequenced. Colonies picked from the last panning rounds were grown overnight by incubating them at 37 °C, 230 rpm in 5 mL of a solution 2TY supplemented with100 μg $mL^{-1}$ carbenicillin. The tubes containing the growth cell cultures were then centrifuged (4100 rpm, 15 min) and the supernatant solution discarded. The cell pellet obtained from this step was treated using a QIAprep® Spin Miniprep Kit (50) according to manufacturer's instructions in order to isolate the phagemid in nuclease-free (NF) $H_2O$ such that it could be sequenced.

## 2.4 Subcloning Affimer Proteins from the Phagemid Vector into pET11a Plasmid

***Transformation of plasmid pET11a into XL1-Blue supercompetent cells and purification.*** 1 μg of DNA pET11a plasmid was mixed with 10 μL of XL1-Blue Supercompetent cells (Agilent Technologies), and the mixture was kept in ice for 30 min. A heat shock was applied by incubating the tubes in a water bath at 42 °C for 45 seconds and, then ice for 2 min. The solution was mixed with 190 μL of LB medium and kept in an orbital incubator at 37 °C and 230 rpm for 1 hour. 100 μL of solution were then plated on LB carb plates (LB agar plates supplemented with 100 μg $mL^{-1}$ carbenicillin). The plates were then incubated overnight at 37 °C. Colonies were picked from each plate and incubated overnight in 3 mL of LB supplemented

with 100 μg $mL^{-1}$ of carbenicillin (37 °C, 230 rpm). The DNA was then extracted by using a QIAprep® Spin Miniprep Kit (QIAGEN), according to the manufacturer's instructions.

***Digestion of pET11a plasmid and pDHIIs phagemid with NotI-HF™ and NheI-HF™ restriction enzymes and ligation.*** Purified pET11a plasmid was digested overnight with NheI-HF™ and NotI-HF™ (20000 units $mL^{-1}$, NEB) enzymes with the addition of 1 μL of Antartic Phosphatase (5000 units $mL^{-1}$, NEB) to 125 μL of the overnight-digested product. The plasmid vector was then purified using NucleoSpin® Gel and PCR Clean-up kit (Macherey-Nagel) according to the manufacturer's instructions. The same operation was conducted with the purified pDHIIs phagemid carrying the Affimer gene. The vector and the insert were then ligated using T4 DNA Ligase (400000 units $mL^{-1}$, NEB) and transformed into XL1-Blue supercompetent cells (Agilent Technologies). The ligated plasmid DNA was then purified using a QIAprep® Spin Miniprep Kit (QIAGEN), according to the manufacturer's instructions and sequenced by Genewiz® through the Sanger method with T7P primer.

## 2.5 Expression and Purification of Affimer Proteins

***Transformation of plasmid pET11a into BL21Star™ (DE3) E. coli.*** pET11a subcloned plasmid was mixed with 10 μL BL21Star™ (DE3) chemically competent *E. coli* cells (Life Technologies), keeping the mixture on ice for 30 min. A heat shock was applied by incubating the solution in a water bath at 42 °C for 45 seconds and, after that, keeping the tubes on ice for 2 min. The solution was mixed with 190 μL of LB medium and incubated at 37 °C, 230 rpm for 1 hour. 100 μL of solution were then plated on LB carb plates (LB agar plates supplemented with 100 μg $mL^{-1}$ carbenicillin). The plates were then incubated overnight at 37 °C.

***IPTG-induced expression of the Affimer proteins.*** The transformants from the plates were grown overnight at 37 °C and 230 rpm in a solution comprising LB supplemented with 100 μg $mL^{-1}$ carbenicillin supplemented with 1% D-glucose. The cell culture was added to a pre-warmed LB solution and grown until an $OD_{600}$ ~ 0.8 was achieved. The cultures were then induced by the addition of IPTG 1M to a final concentration of 0.5 mM. The cell cultures were harvested by centrifugation at 4100*g* for 15 min in a Heraeus Multifuge X3 FR centrifuge. The His-tagged proteins were then purified through Ni-NTA resin (Expedeon), using the wash and the elution solutions described in SI section 1.1. Finally, the purity of the samples was assessed by performing electrophoresis of the eluted Affimer proteins on 15% sodium dodecyl sulfate polyacrylamide gel (SDS-PAGE).

### **2.6** ELISA Assay

An ELISA assay was performed to assess the binding affinity of the Affimer proteins and a control against calcite and aragonite crystals. 1 μM solutions of each protein in 2x Casein Blocking Buffer were added to ≈ 1 mg of calcite or aragonite crystals in 1.5 mL-microcentrifuge tubes and incubated at room temperature on a rotating KingFisher robot (Thermo) for one hour, in order to allow for a more homogeneous mixing. The tubes were then centrifuged at 8000 rpm for 1 min, and the supernatant was removed. The crystals were then washed three times with 500 μL of PBST. After the last wash, 80 μL of a 1:10000 dilution in 2x Casein Blocking Buffer of a HRP Anti-6X His tag® antibody were added to the crystals in the tubes and incubated for another hour on a rotating KingFisher robot (Thermo). After 1 hour, the tubes were centrifuged (3 min, 8000 rpm), and the crystals were washed by discarding the supernatant and replacing with 500 μL of PBST. This operation was repeated for 6 times. After that, the supernatant was discarded and replaced with 50 μL of TMB (3,3′,5,5′-Tetramethylbenzidine).

and incubated at room temperature for 2 min. The tubes were centrifuged (1 min, 8000 rpm) and the supernatant transferred to a Nunc 96-wells plate. The absorbance was read at 620 nm.

### 2.7 Calcium Carbonate Precipitation in the Presence of Affimer Proteins

Calcium carbonate was precipitated using the ammonia diffusion method.[4] 0.5 × 0.5 cm glass slides were cleaned with Piranha solution and immediately before use were cleaned with EtOH and water, and then dried under flowing air. They were then cleaned for 2 min with a Harrick Plasma Cleaner in high plasma mode. The slides were then placed at the base of polypropylene well plates (Nunc) and the wells were filled with a 2 mM solution of $CaCl_2{\cdot}2H_2O$ containing [Affimer] = 10 μM. The well plates were placed open in a sealed chamber with ≈ 2 g of $(NH_4)_2CO_3$ and were left overnight at room temperature. The glass slides were then rinsed with EtOH and MilliQ water and dried under air flow. Experiments were also conducted in the presence of additional magnesium ions, $[MgCl_2{\cdot}6H_2O]$ = 2 mM, and control experiments were performed in the absence of protein.

### 2.8 Calcium Carbonate Precipitation in the Presence of Peptides

Calcium carbonate was precipitated using the ammonia diffusion method in the presence of peptides, in order to verify the importance of the protein conformation upon binding to the crystal surface. The experiments were done as previously mentioned for the $CaCO_3$ precipitation conducted in the presence of proteins. Briefly, 0.5 × 0.5 cm glass slides were cleaned with Piranha solution and immediately before use were cleaned with EtOH and water, and then dried under flowing air. They were then cleaned for 2 min with a Harrick Plasma Cleaner in high plasma mode. The slides were then placed at the base of polypropylene well plates (Nunc) and the wells were filled with a 2 mM solution of $CaCl_2{\cdot}H_2O$ containing [peptide]

= 10 μM, from the protein variable regions. Experiments were conducted testing the two peptides separately and together. The well plates were placed open in a sealed chamber with ≈ 2 g of $(NH_4)_2CO_3$ and were left overnight at room temperature. The glass slides were then rinsed with EtOH and MilliQ water and dried under air flow. Experiments were also conducted in the presence of additional magnesium ions, $[MgCl_2{\cdot}6H_2O]$ = 2 mM. The peptides were purchased from Biomatik at ~1.8 mg and resuspended in DI $H_2O$ to a concentration of 100 μM.

## 3. Characterisation

The crystals precipitated in the presence of the Affimer proteins were characterised using a range of techniques. The polymorphs of the crystals were identified using Raman spectroscopy. Crystals supported on glass slides were analysed with Raman Microscopy using a Renishaw inVia Raman Microscope operating with a 785 nm laser. The morphologies of the crystals were determined using scanning electron microscopy (SEM) conducted with an FEI NanoSEM Nova 450 operating at 5 kV. The glass slides supporting the crystals were mounted on aluminium stubs with double sided Cu tape and were coated with ≈ 2 nm Ir prior to analysis using a Cressington 208HR High resolution Sputter Coater. Focused Ion Beam (FIB) milling was carried out using an FEI Helio G4 CX dual beam, high resolution monochromated FEG SEM instrument. A selected area of the crystals was pre-coated with 2 nm Pt. The operating voltage was 30 kV and the Ga-ion currents were varied between 2.5 nA and 7 nA.

## 4 Molecular Dynamics Simulations

MD simulations were used to investigate the conformational freedom of the variable regions in the Affimer scaffolds in a solution environment for CBA-1, ABA-1 and ABA-2. CBA-2 was excluded due to the longer first-loop region. To perform the MD simulations, input files for each

protein were generated in CHARMM-GUI[5] from the k3 PDB file of the RCSB protein data bank (K3 from the 6YXW complex). All three sequences were trimmed, to remove the His-tag and match the length of the k3 reference (89 residues), with loop residues mutated to match those of the ABA-1, ABA-2 or CBA-1 sequence. The protein was solvated in an octahedral water box (TIP3P water model) with a minimum distance of 10 Å from the protein to the box edge. KCl was added to neutralise and give a final concentration of 0.15 M. The simulations were performed with NAMD.[6] Each system was initially energy minimized (10000 steps) and then heated from 0 to 300 K, whilst applying restraints to the heavy atoms, followed by a short equilibration period at 300 K run for 10000 steps using a timestep of 2 fs. Production simulations were subsequently performed at 300 K, with trajectory frames recorded every 50000 steps (10 ps) for >1750 ns. Simulation trajectories were analysed using Wordom.[7, 8] The first 200 ns of the simulations were omitted from the secondary structure, RMSF and distance histogram analyses in an effort to remove the contribution from the initial state. Secondary structure assignments were achieved using the DCLIKE option in Wordom. Clustering of structures from the whole trajectory was achieved using a RMSD cut-off of 3 Å between clusters.

| Sample | Sequence | pI |
|---|---|---|
| st loop$^{ABA-1}$ | QKKAYMMLDRT | 10.78 |
| 2nd loop$^{ABA-1}$ | KARFWKRYHRN | 12.43 |
| 1st loop$^{ABA-2}$ | QALFHRYRWVT | 12.11 |
| 2nd loop$^{ABA-2}$ | KKKSRMWHEWN | 11.67 |
| 1st loop$^{CBA-1}$ | QGKHKKYFMKT | 11.19 |
| 2nd loop$^{CBA-1}$ | KPFYSKLKTMN | 11.04 |
| 1st loop$^{CBA-2}$ | QAHHRARIRFKVLKT | 14 |
| 2nd loop$^{CBA-2}$ | KQMKYMNSKIN | 11.04 |

**Table S1** Table summarizing the sequences of the peptides tested in mineralization solution, where the terminology used gives the loop and Affimer protein from which the sequence was taken. Isoelectric point (pI) of the peptides was calculated with Innovagen PepCalc.com - Peptide property calculator. The peptides were ordered from Biomatik with modification (acetylation of the N-terminus and amidation of the C-terminus).

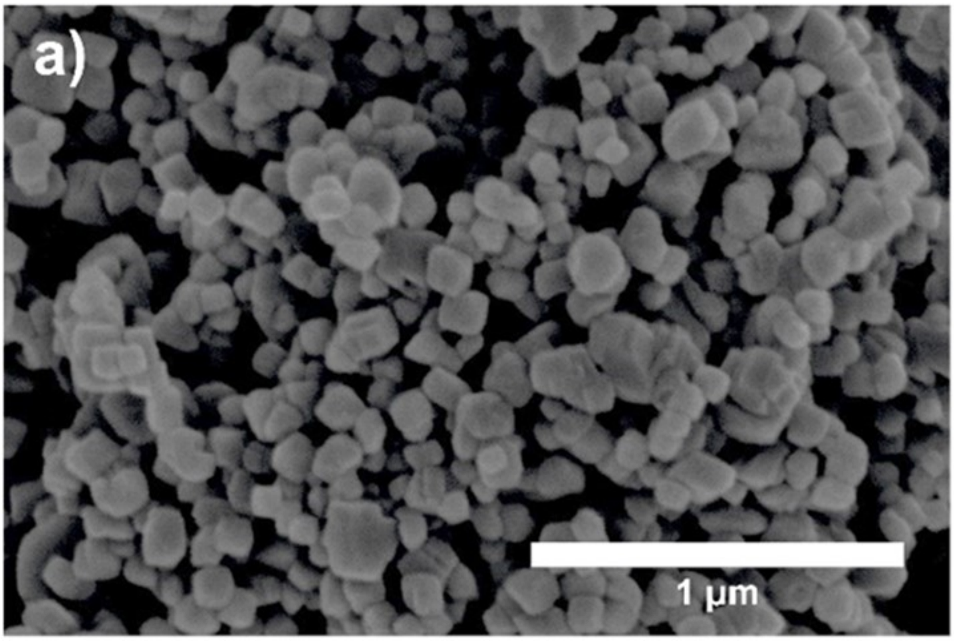


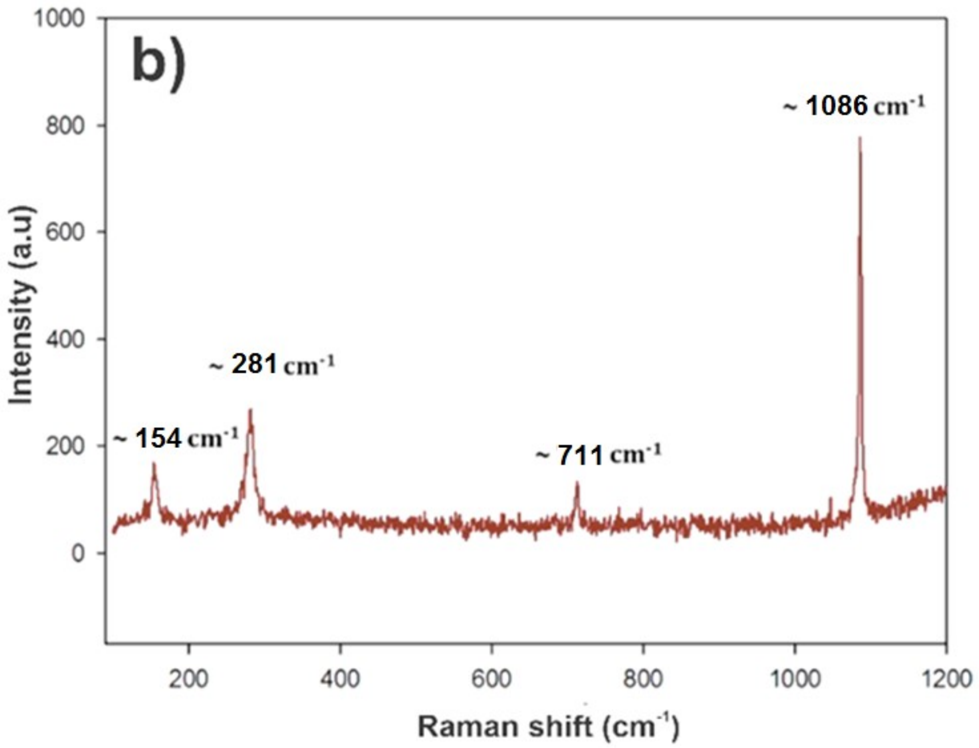


**Figure S1.** Characterization of nano-sized calcite synthesized using the carbonation method. (a) SEM micrographs and (b) Raman spectrum. The graphs show the characteristic peaks of calcite at 154 $cm^{-1}$, 281 $cm^{-1}$, 711 $cm^{-1}$ and 1086 $cm^{-1}$.

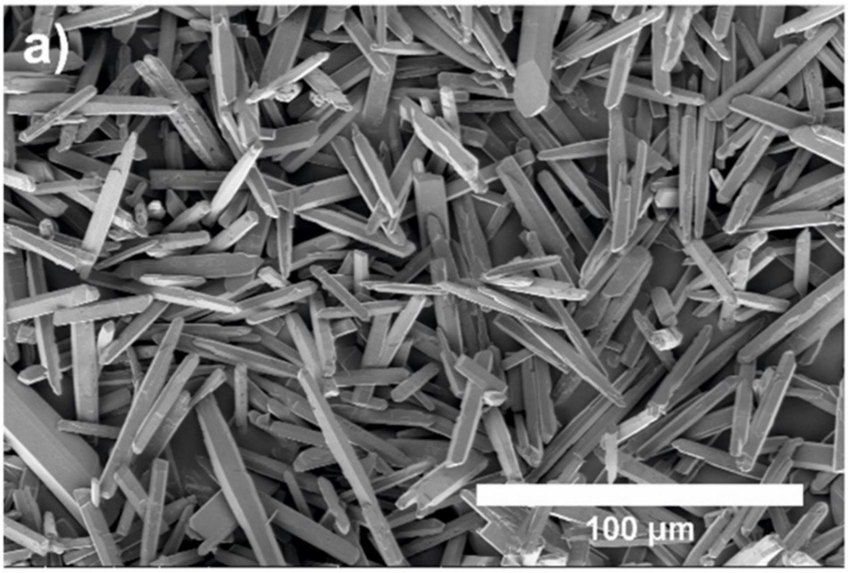


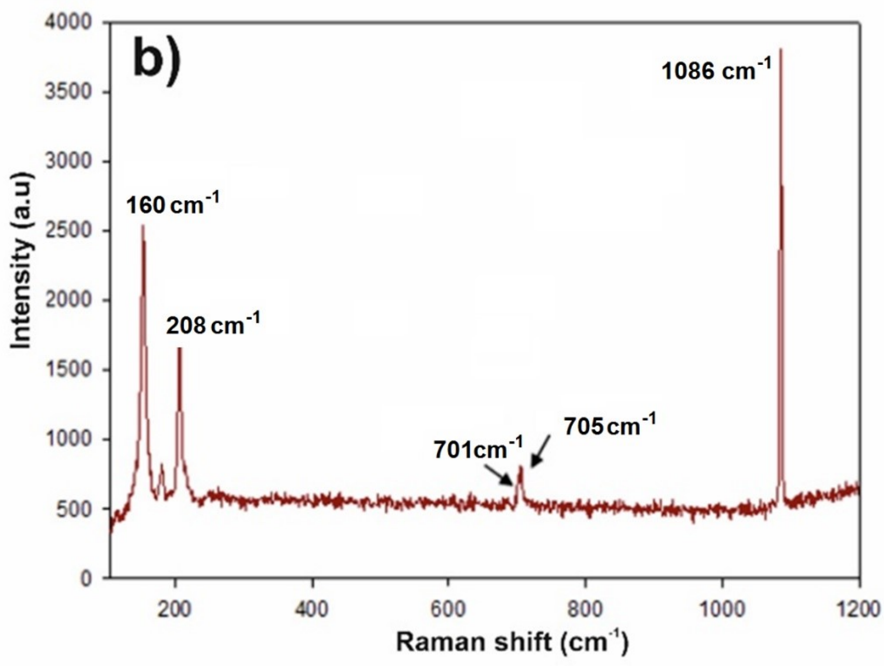


**Figure S2.** Characterization of micron-sized aragonite crystals used in the experiments. SEM micrographs showing uniform needle-like aragonite crystals (a). The crystals present a quite uniform size distribution with a general length of 50–100 μm. (b) Raman spectrum showing the characteristic peaks at 160 $cm^{-1}$, 208 $cm^{-1}$, 701 $cm^{-1}$, 705 $cm^{-1}$ and 1086 $cm^{-1}$: the symmetric stretching $v_1$ at 1086 $cm^{-1}$, the in-plane bending at 701 and 705 $cm^{-1}$ and the peak at 208 $cm^{-1}$ of the translation and libration modes of the $CO_3^{2-}$ ion.[9, 10]

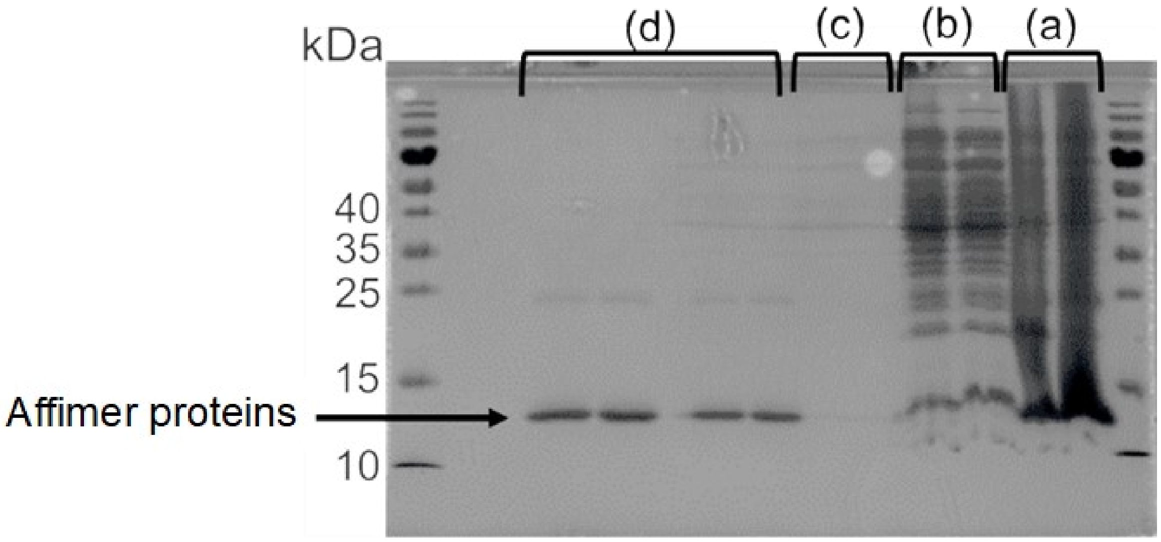


**Figure S3.** Purification profile after denaturing SDS-PAGE analysis. (a) total lysate, (b) soluble fraction, (c) flow-through before elution, (d) elution of ABA-1, ABA-2, CBA-2 and CBA-1 proteins (from left to right). The bands indicated a good expression and purification of the Affimer proteins.

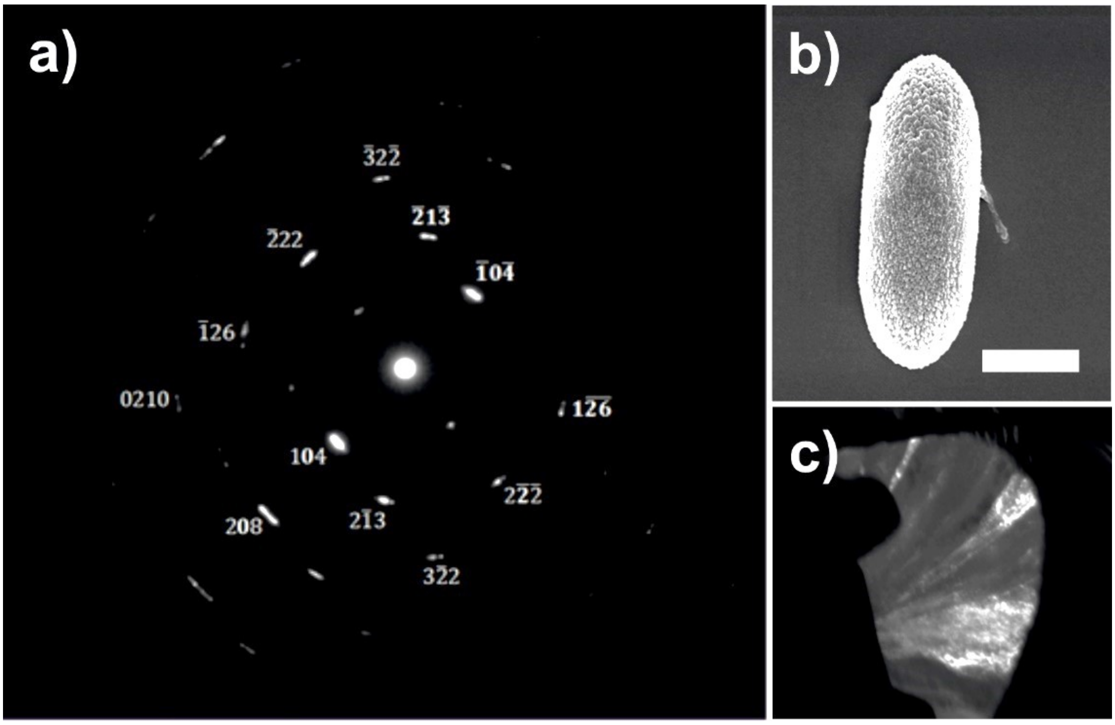


**Figure S4**. (a) Electron diffraction pattern, (b) SEM image and (c) thin section prepared by FIB of the Mg-calcite crystal obtained in the presence of CBA-1 and CBA-2 proteins and magnesium. The diffraction pattern confirmed the single crystallinity of calcite. The curvature of the spots is probably related to the crystal not being a perfect single crystal due to the presence of magnesium ions. Scale bar 3 µm.

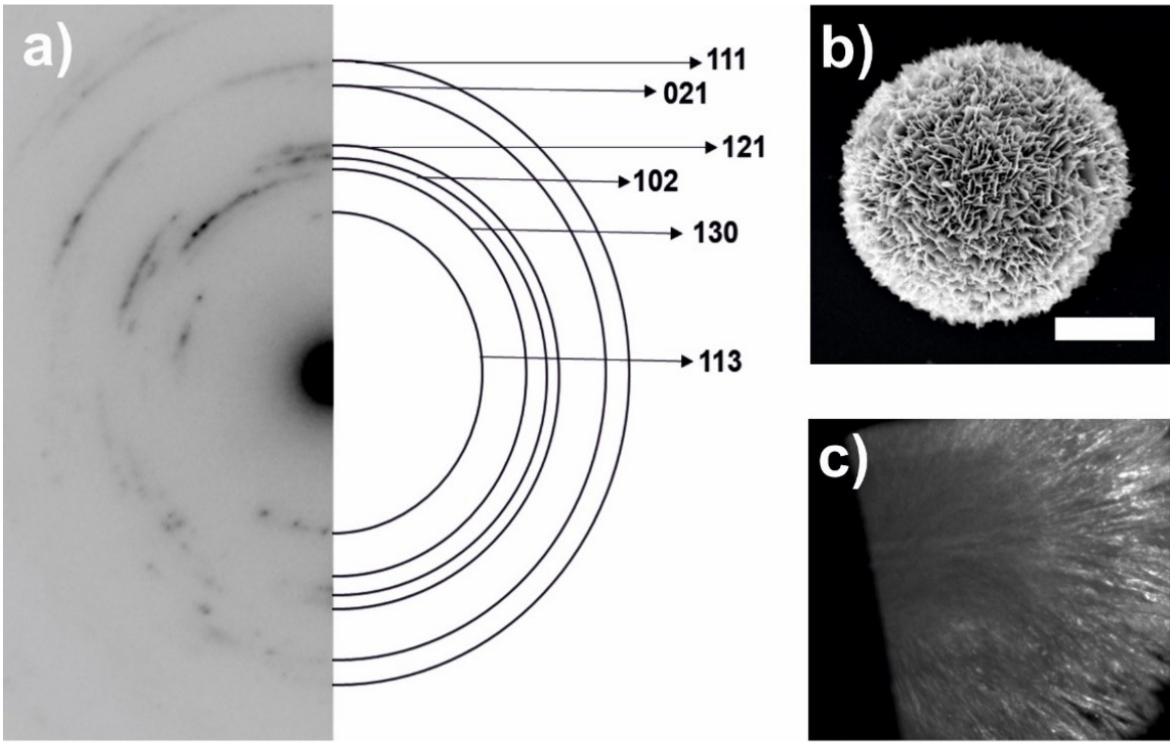


**Figure S5.** (a) Electron diffraction pattern, (b) SEM image and (c) thin section prepared by FIB of the crystals obtained in the presence of ABA-1 and ABA-2 proteins and magnesium. The diffraction pattern confirms the polycrystalline nature of the aragonite crystal. Scale bar 3 μm.

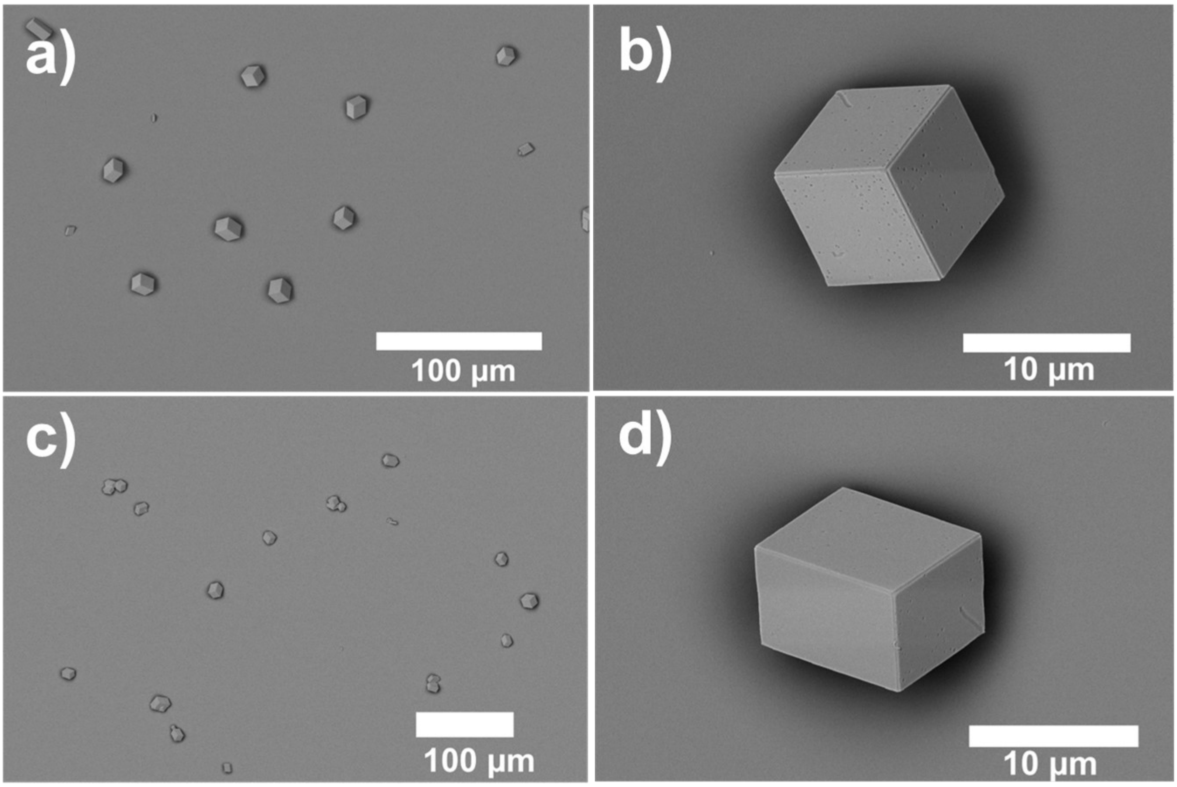


**Figure S6**. SEM micrographs of the crystals obtained using the ADM method in the presence of the peptides (a and b) 1st loop$^{CBA-1}$ and (c and d) 2nd loop$^{CBA-1}$. 10 µM peptides were employed throughout. The same outcome was observed for the other peptides tested.

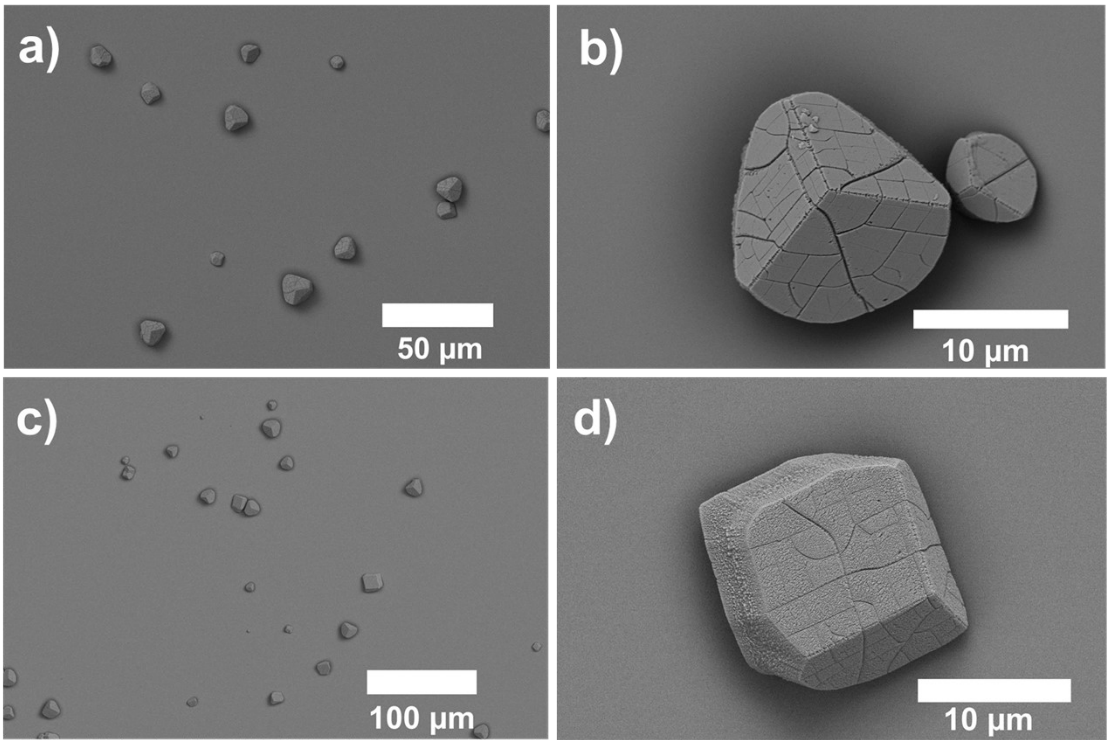


**Figure S7**. SEM micrographs of the crystals obtained using ADM method in the presence of magnesium ions and the peptides (a and b) 1st loop$^{CBA-1}$ and (c and d) 2nd loop$^{CBA-1}$. [$CaCl_2$] = [$MgCl_2$] = 20 mM and 10 µM peptides were employed. The same outcome was observed for the other peptides tested.

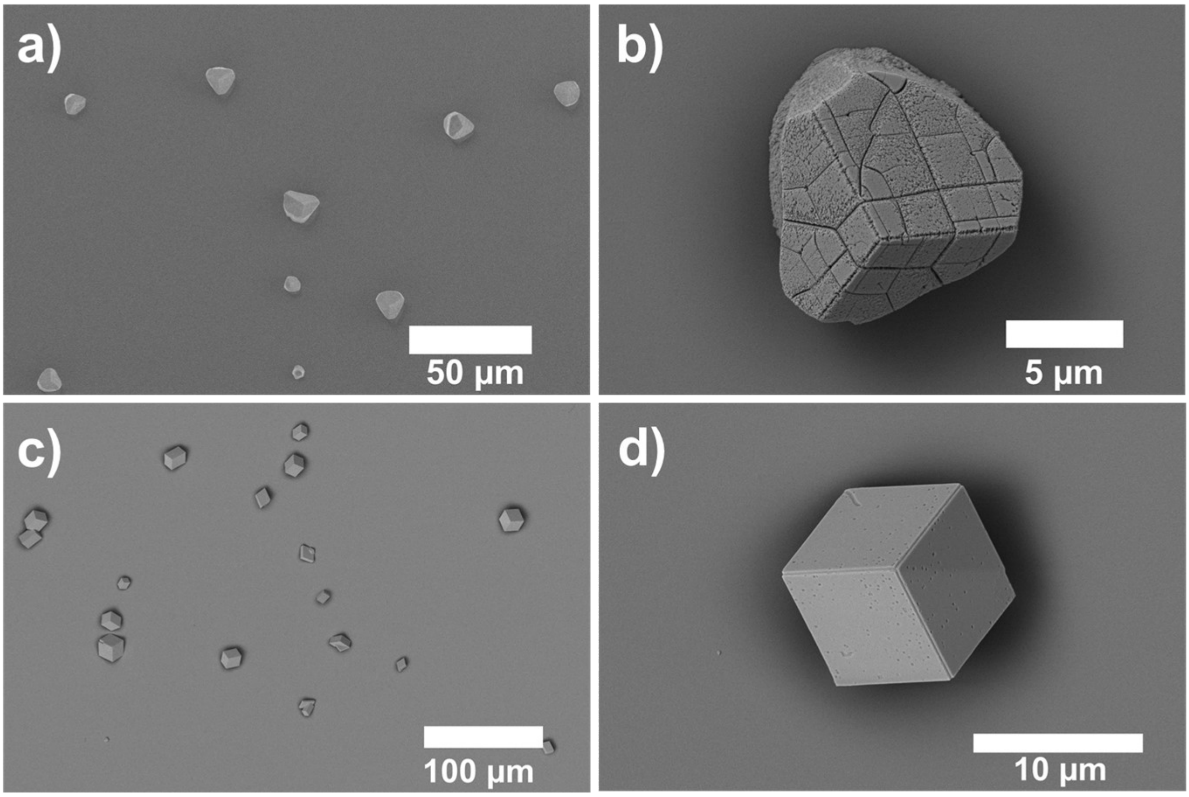


**Figure S8**. SEM micrographs of the crystals obtained using ADM method in the presence of equal concentrations of the peptides 1st loop$^{CBA-1}$ and 2nd loop$^{CBA-1}$ and (a and b) magnesium ions with $[CaCl_2]$ = $[MgCl_2]$ = 20 mM and (c and d) no magnesium ions. The total concentration of peptides in each solution was 10 µM. The same outcome was observed for the other peptides tested.

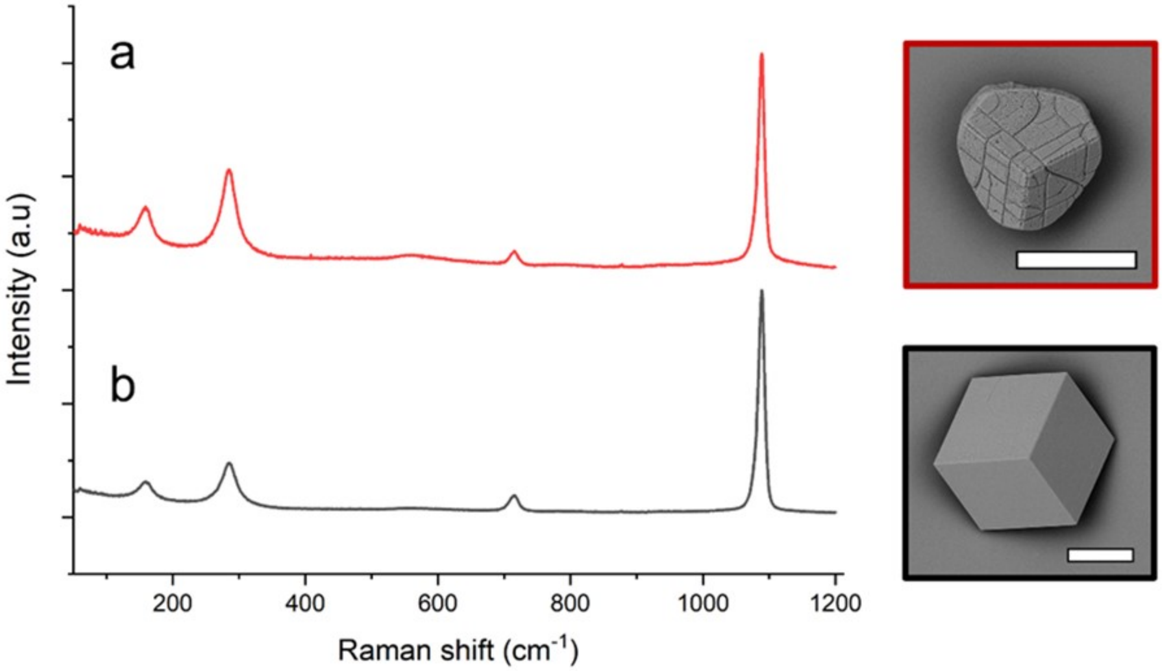


**Figure S9**. Raman spectra of the two kinds of crystals grown in the presence of peptides based on the loop sequences of CBA-1 (insets show SEM images). (a) in the presence of CBA-1 peptides and Ca:Mg = 1:1 and (b) in the absence of magnesium. Both crystals were confirmed to be calcite, with the characteristic peaks at ~ 1086 $cm^{-1}$, 711 $cm^{-1}$ and 281 $cm^{-1}$.

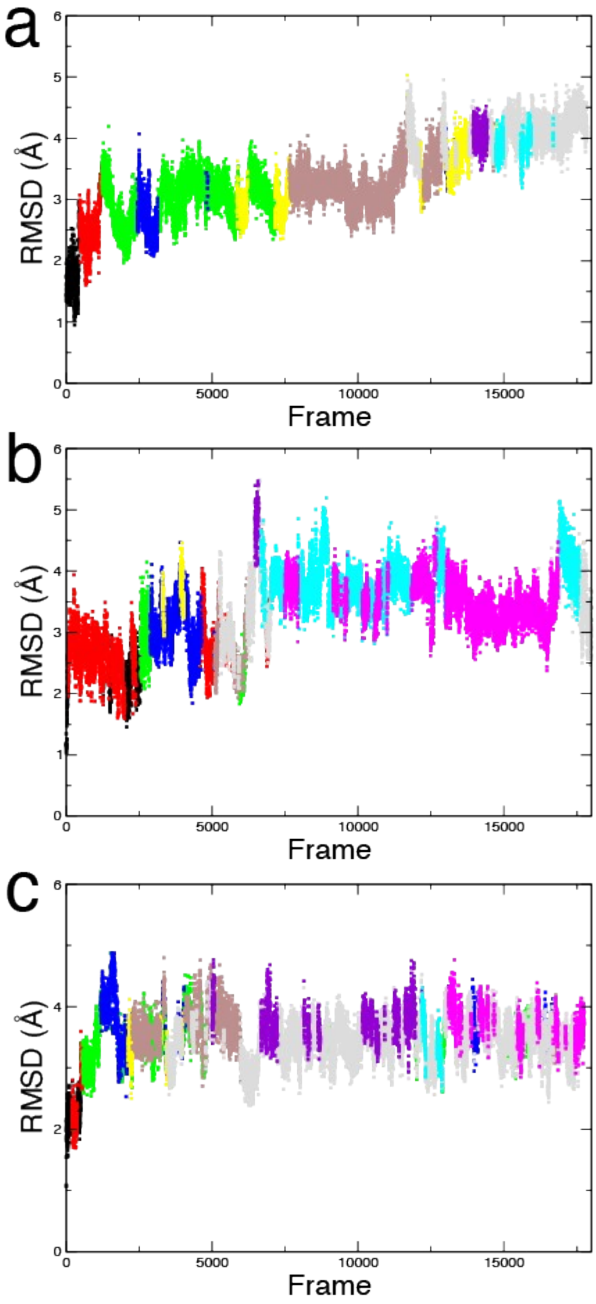


**Figure S10.** Whole protein Cα RMSD values for ABA-1 (a), ABA-2 (b) and CBA-1 (c). The RMSD value for each frame is coloured to show where structures belonging to each cluster were observed as a function of time through the simulation (relative RMSD cluster cutoff of 3 Å). Beyond the early stages of the simulation, reversible transitions between different clusters are observed.

## References


1. D. C. Green, J. Ihli, P. D. Thornton, M. A. Holden, B. Marzec, Y. Y. Kim, A. N. Kulak, M. A. Levenstein, C. Tang, C. Lynch, S. E. D. Webb, C. J. Tynan and F. C. Meldrum, *Nat. Commun.*, 2016, **7**, 13524.
2. L. F. Wang, I. Sondi and E. Matijevic, *J. Coll. Int. Sci.*, 1999, **218**, 545-553.
3. C. Tiede, A. A. S. Tang, S. E. Deacon, U. Mandal, J. E. Nettleship, R. L. Owen, S. E. George, D. J. Harrison, R. J. Owens, D. C. Tomlinson and M. J. McPherson, *Protein Eng. Des. Sel.*, 2014, **27**, 145-155.
4. J. Ihli, P. Bots, A. Kulak, L. G. Benning and F. C. Meldrum, *Adv. Func. Mater.*, 2013, **23**, 1965-1973.
5. B. R. Brooks, R. E. Bruccoleri, B. D. Olafson, D. J. States, S. Swaminathan and M. Karplus, *J. Comp. Chem.*, 1983, **4**, 187-217.
6. L. Kale, R. Skeel, M. Bhandarkar, R. Brunner, A. Gursoy, N. Krawetz, J. Phillips, A. Shinozaki, K. Varadarajan and K. Schulten, *J. Comp. Phys.*, 1999, **151**, 283-312.
7. M. Seeber, M. Cecchini, F. Rao, G. Settanni and A. Caflisch, *Bioinformatics*, 2007, **23**, 2625-2627.
8. M. Seeber, A. Felline, F. Raimondi, S. Muff, R. Friedman, F. Rao, A. Caflisch and F. Fanelli, *J. Comp. Chem.*, 2011, **32**, 1183-1194.
9. P. Gillet, C. Biellmann, B. Reynard and P. McMillan, *Phys. Chem. Mineral*, 1993, **20**, 1-18.
10. M. De La Pierre, C. Carteret, L. Maschio, E. Andre, R. Orlando and R. Dovesi, *J. Chem. Phys.*, 2014, **140**, 164509.
